# Study of the Relationship between *MMP-2* and *MMP-9 *and *Her2/neu* Overexpression in Gastric Cancer: Clinico-Pathological Correlations

**DOI:** 10.31557/APJCP.2021.22.3.811

**Published:** 2021-03

**Authors:** Elham Jafari, Somaye Safinejad, Shahriar Dabiri, Ahmad Naghibzadeh-Tahami

**Affiliations:** 1 *Pathology and Stem Cells Research Center, Department of Pathology, Kerman University of Medical Science, Kerman, Iran. *; 2 *Health Services Management Research Center, Institute for Futures Studies in Health, Kerman University of Medical Sciences, Kerman, Iran. *

**Keywords:** Matrix metalloproteinase, MMP-2, MMP-9, HER-2, gastric cancer

## Abstract

**Background:**

The relationship between the expressions of matrix metalloproteinases with clinico-pathological data on gastric cancer has been investigated in many countries, but this relationship remains unexplored in Iranian patients. Also, the correlation of the MMPs and the HER-2/neu proto-oncogene with other clinic-pathological variables has been evaluated for several other malignancies, but little effort has been made to shed light on the relationship with gastric cancer.

**Methods:**

We investigated MMP-2 and MMP-9 expression and HERE-2/neu overexpression in 48 gastric cancer patients referred to Afzalipour Hospital, associated with Kerman Medical University. Immunohistochemistry staining with rabbit polyclonal antibodies was used. Data statistical analysis was done by SPSS software (Version 20.0).

**Results:**

The mean age was 59, most of the patients were male (79.2%), and the average tumor size was larger than 5 centimeters in its greatest diameter. The majority of tumors were of the intestinal subtype and were located in the pyloric and antrum regions (43.8%). Invasion to muscularis properia was seen in 87.5% of the tumors (T3). MMP-2 and MMP-9 were highly expressed in 58.3% and 50% of cases, respectively, and Her-2/neu positivity was 10.4%. MMP-2, MMP-9 and HER-2 were found to have no relation with any clinicopathological parameters.

**Conclusion:**

According to the results of this study, MMP-2 and MMP-9 were highly expressed in gastric cancer, but there was no significant association with other clinicopathological variables.

## Introduction

According to GLOBOCAN’s (Global Cancer Observatory) 2018 data, although gastric cancer incidence has decreased in recent years, gastric cancer is still the third leading cause of death from cancer worldwide (Bray et al., 2018). Annually, more than one million new cases are diagnosed (Bray et al., 2018), 110,000 of whom belong to Iran (Zendehdel, 2019). Metastasis is the most important factor behind the high mortality in patients suffering from gastric cancer (Oo et al., 2014). Two essential steps in metastasis are migration and invasion of tumoral cells, which consist of several interconnected steps including proliferation, detachment, circulation, transport, arrest in organs, adherence to the vessel wall, extravasation, the establishment of a microenvironment, and proliferation in distant organs (Li et al., 2014). 

Matrix metalloproteinases (MMPs) play an essential role in tumor growth, angiogenesis, and metastasis. They are a large family of zinc-dependent proteolytic enzymes that protelyse the extracellular matrix (ECM), regulate angiogenic factors, cleave E-cadherin to adjust the interactions between tumor cells, and process integrins to adjust the interaction between tumor cells and ECM, thus increasing the invasiveness of tumor cells (Nelson et al., 2000; Roy et al., 2009). Two key members of the MMP family are matrix metalloproteinase-2 (MMP-2, 72-kDa gelatinase, called type IV collagenase) and matrix metalloproteinase-9 (MMP-9, 92-kDa gelatinase, called type IV collagenases) (Sheu et al., 2003; Odaka et al., 2005). The overexpression of these two markers has been reported in several tumors such as ovarian cancer, cervical cancer, hepatocellular cancer, head, neck, and thyroid carcinomas, squamous cell carcinomas, and gastric cancer. MMP-2 and MMP-9 have also been associated with tumor stage, lymphatic invasion, lymph node metastasis, and recurrence (Wu et al., 2006; Roy et al., 2009; Chu et al., 2011).

The HER family includes four plasma membrane-bound receptors. The most important member is the human epidermal growth factor receptor-2 (HER-2/neu, a proto-oncogene encoded by ERBB2 located on chromosome 17. HER2 is expressed in many tissues, including the breast, the gastrointestinal tract, the kidneys, and the heart. It plays a major role in tumorigenesis by promoting cell proliferation and suppressing apoptosis, facilitating excessive/uncontrolled cell growth and cancerization (Neve et al., 2001; Rubin and Yarden, 2001; Ménard et al., 2003). HER2 gene amplification was first detected in breast cancer and is significantly associated with a worse prognosis (Slamon et al., 1987).

Amplification of HER2 is also present in several other malignancies such as colorectal cancer, ovarian cancer, prostate cancer, lung cancer, and, particularly, gastric and gastroesophageal cancers (Yan et al., 2015). The frequency of HER2 overexpression in gastric and gastroesophageal cancer is very different in studies and has led to conflicting results concerning her-2 prognostic value (Janjigian et al., 2012; He et al., 2013; Baykara et al., 2015). Recently, trastuzumab (anti-HER-2polyclonal antibody) has been used to treat patients with advanced gastric cancer, leading to a rapid increase in the clinical demand for HER2 assessment.

Also, target therapy with anti MMP-2 and anti MMP-9, as anti-metastatic agents, has been evaluated in some clinical trials (Webb et al., 2017). Due to the great potential of these three markers (HER-2, MMP-2, and MMP-9) in the prognosis and treatment of advanced stages of gastric cancer, and the absence of investigations directly comparing MMP-2 andMMP-9 with HER-2 expression, we decided to evaluate this subject. To the best of our knowledge, this is the first study to assess the expression of matrix metalloproteinases in Iranian patients suffering from gastric cancer.

## Materials and Methods

*Patients*

This cross-sectional study was conducted among 48 patients undergoing gastrectomy due to gastric cancer at Afzalipour Hospital in Kerman between January 2014 and December 2017. Demographic data was initially collected from the Cancer Registry Center of Kerman to identify the gastric cancer patients, and samples were then obtained from Afzalipour Hospital pathology lab. A pathologist reassessed the prepared slides to identify the type of tumor, stage, histologic grade, invasion to peripheral tissues and lymph nodes, vascular invasion, presence of helicobacter pylori, intestinal metaplasia, and atrophy.

Tumor grade and stage were classified according to the eighth edition of the American Joint Committee on Cancer (AJCC) staging manual’s tumor-node-metastasis (TNM) classification (Hadi et al., 2016).

Tumor types were divided into intestinal, diffuse, and mixed according to the Lauren Classification (Seo et al., 2017). Tumor locations were divided into cardia/fundus, corpus, antrum, and pyloric, according to Sreeram et al. (Sreeram et al., 2017). Histological grades were classified as G1 (well-differentiated), G2 (moderately differentiated), G3 (poorly differentiated or undifferentiated).

None of the patients had received chemotherapy or radiotherapy before the surgery, and no additional malignancies were evident. The study was approved by the ethics committee of the Medical faculty of Kerman Medical University.

*Immunohistochemistry*

 After marking a suitable site (non-necrotizing tumor) on HandE slides, the paraffin-embedded tissues were sectioned at a 3–4 micrometer thickness then stained using the IHC method with HER-2/Neu Rabbit polyclonal antibody (Biogenax kit) and MMP-2 and MMP-9 Rabbit polyclonal antibody (Zytomed, Germany). The slides were then observed with an Olympus microscope (model CX33, Japan) at ×400 and ×100 amplification to determine the staining score. Matrix metalloproteinase was not stained in normal gastric mucosa.

*Scoring system*

**Figure 1:**
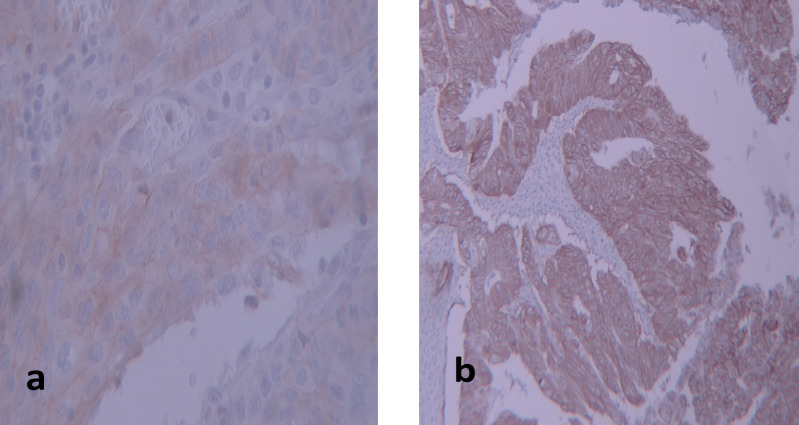
Images of Immunostaining for Her2-neu Expression. a, weakly positive/equivocal: grade +2 (magnification×400 ); b, strong positive: grade +3 (magnification ×100 )

For HER-2 /neu, all sides were scored according to the DAKO criteria from 0 to 3+ (Figure 1).

The DAKO criteria for HER-2/Neu include 0: no membranous staining for HER-2/Neu or lower than 10% staining of tumoral cells are scored as negative expression; 1+: incomplete membranous staining or staining in higher than 10% of tumoral cells; expression of HER-2/Neu is negative; 2+: complete membranous staining at mild to moderate degree in more than 10% of tumoral cells (these are reported as borderline and should be certified with FISh/CISH methods); 3+: complete membranous staining in 10% of tumoral cells showing positive HER-2/Neu expression (Figure 1). 

MMP-2 and MMP-9 staining were evaluated by a Rajkumar score (Rajkumar et al., 1996), in which the staining intensity was scored as follows: score 0 (negative staining), 1 (weak staining), 2 (moderate staining), and score 3 (intense staining) in at least five different high-power fields. Based on the number of positive tumor cells, the staining was scored as follows: “0” if 0%–10% of the tumor cells were positively stained, and “1” if 11%–25%, “2” if 26%–50%, “3” if 51%–75%, and “4” if 76–100% of the cells were positive.

**Figure 2:**
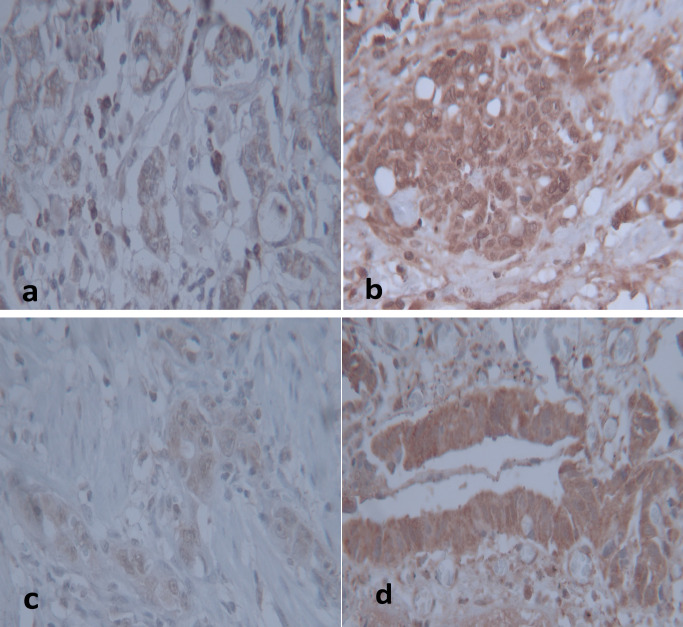
Images of MMP-2 and MMP-9 protein expression in gastric cancer tissues by immunohistochemistry (magnification×400). a, Low expression of MMP-2; b, High expression of MMP-2, c) Low expression of MMP-9, d) High expression of MMP-9

The final score was based on the multiplying of the staining extent and intensity scores. The slides scored 8 or higher were classified as high expression (positive), and others with less than 8 as low expression (negative)(Figure 2). 

*Statistical analysis*

The association between MMP-2 and MMP-9 expression and clinicopathological variables was tested using a chi-square test. Data analysis was done by SPSS software (version 20.0).

## Results

*Clinicopathological characteristics*

The demographic data of patients are shown in Table 1. The median age was 59 (ranging from 25 to 83). Most of the patients were male (72.9%), with tumors larger than 5 centimeters in size (54%), intestinal-type (72.9%), invading muscularis properia (87.5%), and lymphovascular invasion (91.7%).

*Relationship between clinicopathological variables and MMP-2, MMP-9, and HER-2 overexpression*

Among 48 patients, MMP-2 protein overexpression was seen in 28 cases (58.3%), and MMP-9 was positive in 24 (50%) of them. Also, 5 (10.4%) of the patients were positive for HER-2/neu. In this study, no significant associations were identified between MMP-2, MMP-9, and HER-2/neu overexpression with clinicopathological parameters (Tables 2 and 3). Also, MMP-2 and MMP-9 with HER-2/neu overexpression did not have a meaningful association (Table 4).

**Table 1:** Clinicopathological Variables of Patients

Variables	N (%)	Percent
Age (Years)		
<60	22	45.8
≥60	26	54.1
Sex		
Male	38	79.2
Female	10	20.8
Tumor Location		
Antrum/Pylori	21	43.8
Antrum/Body	6	12.5
Cardia/Fondus	17	35.4
Corpus	4	8.3
Tumor Size (cm)		
< 5	13	27.1
≤5	31	46.6
Undetermined (Diffuse type)	4	8.3
Tumor type		
Intestinal	35	72.9
Diffused	12	25
Mixed	1	2.1
Histological Grade		
Madrate	27	56.3
Poor	21	43.8
Depth of invasion		
T1	4	8.3
T2	1	2.1
T3	42	87.5
T4	1	2.1
Lymphovascular Invasion		
Positive	44	91.7
Negative	4	8.3
Lymph node involvement		
N0	16	33.3
N1	8	16.6
N2	16	33.3
N3	6	12.5
Nx	2	4.1

**Table 2:** Relationship between MMP-2, MMP-9, and HER-2 and Clinical Data

Parameter	MMP-2 Positive		MMP-9 Positive		HER-2 Positive	
	N (%)	P-value	(N%)	P-value	(N%)	P-value
Age(years)						
60 >	12 (42.9)	0.77	14 (58.3)	0.07	1 (25)	0.61
60 ≤	16 (57.1)		10 (47.1)		4 (75)	
Sex						
Male	23 (82.1)	0.7	18 (75)	0.36	4 (75)	0.7
Female	5 (17.9)		6 (25)		1 (25)	
Tumor location						
Antrum/pylor	11 (39.3)	0.19	10 (41.5)	0.107	2 (25)	0.545
Antrum/body	6 (21.4)		3 (12.5)		0	
Cardia/fundus	9 (32.2)		9 (37.5)		3 (75)	
Corpus	2 (7.1)		2 (8.5)		0	
Tumor size						
< 5	10 (38.5)	0.33	8 (33.3)	0.53	0	0.55
≥ 5	16 (61.5)		16 (66.7)		5 (16.1)	

**Table 3:** Relationship between MMP-2, MMP-9, and HER-2 and Pathological Data

Parameter	MMP-2 positive		MMP-9 positive		Her2 positive	
	N (%)	P-value	N (%)	P-value	N (%)	P-value
Tumor type						
Intestinal	21 (75)	0.57	17 (70.8)	0.59	3 (75)	0.93
Diffuse	6 (21.4)		6 (25.0)		2 (25)	
Mixed	1 (3.6)		1 (4.2)		0	
Histologic grade						
Madrate	15 (53.6)	0.44	12 (50.0)	0.28	3 (75)	0.49
poor	13 (46.4)		2 (50.0)		2 (25)	
Depth of invasion						
T1	4 (14.3)	0.17	0 (0.0)	0.1	1 (25)	0.60
T2	1 (3.6)		1 (4.2)		0	
T3	22 (78.6)		22 (91.7)		4 (75)	
T4	1 (3.6)		1 (4.2)		0	
Lymphovascular invasion						
Positive	25 (89.3)	0.48	23 (95.8)	0.29	5 (100)	0.47
Negative	3 (10.7)		1 (4.2)		0	
Lymph node metastasis						
N0	8 (28.6)	0.24	7 (29.2)	0.79	3 (75)	0.20
N1	4 (14.3)		5 (20.8)		0	
N2	12 (42.9)		9 (35.7)		2 (25)	
N3	2 (7.1)		2 (8.3)		0	
Nx	2 (7.1)		1 (4.2)		0	

**Table 4:** Association of MMP-2 and MMP-9 with HER-2/neu Overexpression

Factor	MMP-2		MMP-9	
HER-2	positive	P-value	Positive	P-value
Negative	20 (74.1)	0.24	19 (79.2)	0.37
1+	4 (14.8)		1 (4.2)	
2+	2 (7.4)		1 (4.2)	
3+	1 (3.7)		3 (12.4)	

## Discussion

Gastric cancer is one of the most common malignancies in the world (Bray et al., 2018), and the prognosis of advanced stages is very poor, with a 7.5–12 month survival in treatment by chemotherapy and a 3-5 month survival with the best supportive care (Wagner et al., 2010). As a result, the evaluation of prognostic markers to find a suitable marker for preventing tumor progression is of great importance. For this aim, we evaluated MMP-2 and MMP-9 expression and also HER-2/neu overexpression in 48 gastrectomy specimens using the immunohistochemistry method. Among all the matrix metalloproteinases, MMP-2 and MMP-9 are the most well-known because they can facilitate tumoral cell invasion by degrading type IV collagen, gelatin, and laminin in the basement membrane. As we know, the initial step for invasion and metastasis is destroying the cell-cell and cell-ECM interactions; therefore, MMP-2 and MMP-9 play an essential role in metastasis. Thus, the analysis of these two markers can help to prevent tumor progression using target therapy. 

MMP-2 expression was reported to range from 35% to 73.9% by Shen et al. (Shen et al., 2014), who evaluated ten studies from 1998 to 2013. Our findings also bore this meta-analysis result. Zhang et al., (2012) conducted a meta-analysis of MMP-9 expression in gastric cancer in 11 studies from 1996 to 2010, reporting that MMP-9 expression ranged from 50.7% to 81%. In our study, MMP-9 was positive in 50% of patients, which agrees with Zhang’s et al., (2012) results. 

In our study, most of the patients (87.5%) were in the advanced stage (T3), and MMP-2 and MMP-9 were stained in 78.6% and 91.7% of tumoral specimens, respectively, but the relationship was not significant (p > 0.05). This is similar to Hwang et al. and Lee et al.’s findings (Lee et al., 2008; Hwang et al., 2010), but not in line with the majority of previous studies (Zhang et al., 2003; Wu et al., 2005; Alakus et al., 2008). This may be because of the different scoring systems and the larger sample size than our study.

Among cases with lymphovascular invasion ,89.3% and 95,8%, were positive for MMP-2 and MMP-9, respectively, but unlike Chen et al., (2014) and Zheng et al.,’s (2006) results, the relationship was not significant.

MMP-2 was positive in 75% of patients with intestinal subtype, and also MMP-9 was positive in 70.8% of them, but, unlike Mrena et al., (2006), our results were not significant. This may be because of the large sample size of their study, including 329 gastric adenocarcinoma cases. It is worth mentioning that their scoring system only consisted of a percentage of positive tumor cells, so the intensity of staining did not impact the scores.

In this study, we have not found any relation between MMPs and patient age, sex, tumor location, grading, and lymph node involvement, which is in agreement with Bronschein et al.’s results (Bornschein et al., 2015).

The frequency of HER-2 expression differs in different studies, which have reported 4.4% to 53%.4 expression, with a mean of 17.9% (Slamon et al., 1987; Janjigian et al., 2012; He et al., 2013; Baykara et al., 2015; Yan et al., 2015). The results of the present study show HER-2 expression to be lower than the mean of HER-2 in other studies. This may be due to technical errors or differences in antibody use in IHC staining in this study.

Some studies, similar to ours, have demonstrated no prognostic value in HER-2/neu (Grabsch et al., 2010; Wang et al., 2011; Janjigian et al., 2012; He et al., 2013; HAYATBAKHSH et al., 2019). Some papers have shown that HER-2 induces MMP expression in breast cancer (O-Charoenrat et al., 2002; Pellikainen et al., 2004). They have demonstrated that HER-2’s effect on tumor invasiveness is due to the upregulation of MMP-2 and MMP-9 expression and the regulation of proteolytic activity. O’charoenrat et al. showed that target therapy by MMP inhibitors blocking EGF receptors successfully inhibited tumor progression in head and neck squamous cell carcinomas (O-Charoenrat et al., 2002), but in this study, HER-2 and MMPs did not have a meaningful relationship. This may be due to the low positivity of HER-2 immunostaining in small-size samples.

In conclusion, according to the results of this study, MMP-2 and MMP-9 were highly expressed in gastric cancer, but there is no significant association with other clinicopathologic variables. Further studies with larger sample sizes and equal distribution of samples among all stages of cancer can help assess the relationship of MMPs and HER2 with the prognostic factors of gastric cancer.

## Author Contribution Statement

study conception and design : Elham Jafari and Shahriar Dabiri, data collection: Elham Jafari and Somaye Safinejad, analysis and interpretation of results: Ahmad NaghibzadehTahami , Elham Jafari and Shahriar Dabiri, draft manuscript preparation: Elham Jafari and Somaye Safinejad. All authors reviewed the results and approved the final versions of the manuscript.
